# Removal of Cedar Pencil from Bladder of Railway Engineer

**Published:** 1893-04

**Authors:** H. L. Battle

**Affiliations:** Wadley, Ga.


### REMOVAL OF CEDAR PENCIL FROM BLADDER OF RAILWAY ENGINEER.

---

BY H. L. BATTLE, M. D., WADLEY, GA.

---

On November 3, 1892, P. H. H., age 45, came to my office. His face was deathly pale, and he seemed very greatly excited, so much so that my first impression was that the man was seriously hurt. To my question as to what was the matter, he replied that he had a cedar pencil in his bladder and he wanted me to remove it, and that he only had twenty minutes before leaving for Macon. I had him to remove his pants and drawers, and found the pencil presenting in the perineum. He said it was four or five inches long. Thinking I might remove it through the urethra, I procured a long and slender dental tool from my friend, Dr. E. S. Daniel, a highly accomplished dental surgeon, whose office adjoined my own, I introduced the dental tool and caught the point under the pencil, and made an ineffectual effort to extract it. I then gave the instrument to Dr. Daniel, and introduced my finger in the rectum, but could not reach the end. My object was to press it downward and outward. Failing in this, I informed him the only alternative was to cut it out. He readily consented, but refused to allow me to give either chloroform or ether. I then placed him in the lithotomy position. While my instruments were soaking in alcohol I washed the field of operation and adjoining paits well with soap and water. After wiping it dry, I then sponged it well with a 1 to 200 mercuric solution. With a common bistory I cut through the perineum down to the urethra, making a wound of about one inch. I then made a small cut into the urethra, the point of pencil presented promptly and was caught by Dr. Daniel with a pair of forceps and gently drawn while I slightly enlarged the opening. The pencil measured seven inches long. I then sponged the wound well with a 1 to 3000 bichloride solution, dusted it well with iodoform, put one suture of Sand J.'s twisted silk No. 6, placed over this 2 or 3 folds of bichloride gauze, and over this a piece of guttapercha tissue, and held the dressing in position by two strips of rubber adhesive plaster, crossing each other over the wound in the form of an x. After finishing the dressing, he had two minutes to spare. He went from my office to his engine and started for Macon, 86 miles distant. After arriving in Macon, he violated some rule of the company, and was suspended for fifteen days. I saw him no more until the 21st November, when he again walked into my office. He said he was well—had never stopped a minute on account of it—it had given him no trouble in walking, standing or urinating; that neither his wife or his fireman knew anything about it. He allowed the dressing to remain on for eight days.

I have reported this case, not on account of anything new, either in the operation or dressing, but solely on account of its novelty and the difficulty of passing a stiff cedar pencil, seven inches long, under the pubic arch and into the bladder. This is a mystery to me. I have frequently failed with a catheter, and I am sure I should have made a miserable failure with a pencil.

What was he doing with a pencil in the urethra, anyhow? He says he was lying on his bunk, and after signing his report, and being about half-asleep, he introduced it, and it got away from him and made its way into the bladder. Was he masturbating? His evident disinclination to speak of it; his disconnected history of the case, together with his general excited appearance, lead me to believe that he was.

